# “A person who does not have money does not enter”: a qualitative study on refugee women’s experiences of respectful maternity care

**DOI:** 10.1186/s12884-022-05083-2

**Published:** 2022-10-05

**Authors:** Tamar Kabakian-Khasholian, Jihad Makhoul, Aleni Ghusayni

**Affiliations:** Department of Health Promotion and Community Health, Faculty of Health Sciences, American University of Beirut, Beirut, Lebanon

**Keywords:** Childbirth, Labor, Refugee health, Respectful maternity care, Arab, Lebanon

## Abstract

**Background:**

Women’s childbirth experiences in health facilities is at the core of quality of care. Their perceptions of poor-quality care, including disrespectful care at health facilities during childbirth, is recognized as a significant barrier to seeking care for subsequent births. Research that explores women’s perspectives of the dimensions of disrespect and mistreatment during childbirth in Arab countries is scarce, and there is none pertaining to refugee groups who carry the burden of multiple vulnerabilities and who suffer from discontinued care, especially in fragile health systems. This paper aims at presenting Palestinian, Iraqi and Syrian refugee women’s experiences, understanding and interpretation of disrespect and mistreatment during childbirth in hospitals in Lebanon.

**Methods:**

This study employed phenomenology, a qualitative research design to generate data through in-depth interviews. Women who were 3 to 6 months postpartum were recruited through the non-governmental organizations (NGOs) that are actively engaged in providing welfare and healthcare services to different populations of refugee women in Lebanon. In total, 24 women were interviewed. All interviews were audio recorded, transcribed verbatim and subjected to thematic analysis.

**Results:**

Of the 24 women who participated in this study, 9 were Palestinian, 12 were Syrian and 3 were Iraqi. The participants spoke of restricted choices to hospitals, care providers and to types of birth, while revealing adverse experiences during childbirth in healthcare facilities, including verbal and physical abuse, disrespectful verbal and non-verbal communication by healthcare providers. They also reported sub-standard care, denial of birth companions and breaches to their privacy. Our findings exemplify how the coverage of the cost of facility-based births by UN agencies and NGOs increase refugee women’s vulnerability to disrespect and mistreatment during childbirth.

**Conclusion:**

This study shows how disrespect and mistreatment are intertwined in a complex system which is devised to ensure access to facility-based birth for displaced populations. Implications for programs and policies point to the need for strengthening capacity and for providing resources for the adaptation of global guidelines into context-specific strategies for the provision of quality maternity care during humanitarian crises and beyond.

**Supplementary Information:**

The online version contains supplementary material available at 10.1186/s12884-022-05083-2.

## Introduction

There is growing global recognition of the pivotal role that the provision of respectful maternity care has in improving quality of care for women and newborns [1, 2]. Respectful, dignified, equitable and women-centered care are recognized as key components of good quality maternity care [3, 4]. The WHO framework for quality of maternal and newborn care critically positions the experience of care equally with the provision of safe clinical care in defining quality of care [3]. Considering that provision of effective quality care in health facilities is highly impactful in reducing maternal and neonatal mortality and morbidity [5], having a positive childbirth experience in health facilities is therefore at the core of quality of care.

Disrespect and mistreatment during childbirth have been documented in low-income settings^,^ [1, 6–10], along the continuum of care and include non-dignified care, subtle humiliation of women, discrimination of certain groups and physical and verbal abusive treatment of women. Women’s perception of poor-quality care including disrespectful care at health facilities during childbirth is recognized as a significant barrier to women seeking care for subsequent births [11]. This is particularly important to address for marginalized women, such as refugees who experience multiple vulnerabilities, live in uncertainty and already suffer from discontinued care, especially in fragile health systems.

Social, economic and professional factors defining the interaction between women and providers [7, 12], as well as organizational structures and the failures at the level of health systems largely contribute to disrespectful maternity care [1, 13]. Studies from India [14], Tanzania [15] and Eastern Europe [16], highlight the role of structural violence that manifests in healthcare providers’ perceptions of marginalized groups of women based on gender, age, race, ethnicity and social class. These biases have resulted in the differential treatment of women during pregnancy, labor, birth, and postpartum.

Despite improvements in maternal health outcomes [17], quality of maternity care in the Arab region remains sub-optimal [18] with evidence showing marginalization of women and their needs in the process of care [19–23]. Research from this region points to health system level factors [24] and describes the undesirable experiences of women during childbirth [20–23] indicating dehumanized childbirth care [25]. Studies from Lebanon in specific have documented women’s fears, marginalization and dissatisfaction during facility-based births. A study exploring healthcare providers’ perspectives on the provision and use of family planning services among Syrian refugees in Lebanon highlights the biases in the attitudes of providers that shape service delivery and manifest in discrimination against these population [26]. This is important to consider within the efforts put into recommended emergency response approaches for reproductive health services, which have been found to be deficient in terms of cultural sensitivity towards refugee populations [27].

Lebanon has hosted different groups of refugees throughout its history. Around 400,000 Palestinian refugees came to Lebanon after the start of the Arab-Israeli conflict in 1948. They reside in camps located mainly in the suburbs of Beirut [28]. For Palestinians, education and healthcare services are offered primarily by UNRWA (United Nations Relief and Works Agency for Palestine Refugees in the Near East) and partially through non-governmental agencies (NGOs). Lately, international funding for UNRWA has decreased, affecting the quality and quantity of healthcare services offered. Since 2003, there are over 9,400 Iraqi refugees and asylum seekers scattered all over Lebanon [29] receiving subsidized healthcare and support through UNHCR (United Nations High Commissioner for Refugees) and NGOs. Since 2011, around 1.5 million refugees have come into Lebanon from Syria [30]. Reproductive health services are provided to UNHCR registered refugees at primary healthcare centers, the majority of which are run by NGOs by subsidizing up to 85% of diagnostic tests for pregnant women and 75% of the total cost of childbirth followed by two postnatal visits. The balance is paid either by international or local organizations or out of pocket. Unregistered refugees have access to some services, such as the first antenatal care visit, and care for childhood and maternal acute illnesses [29]. There are few international NGOs that cover the remaining cost of childbirth in hospitals, offer free antenatal care, and even free labor and childbirth services in the few clinics set up for this purpose [31]. The UN agencies and INGOs have established financial coverage agreements with a number of private and public hospitals in Lebanon, a situation that limits the choices of place of birth for refugee women.

The private-public partnership and the response of the UN agencies and civil society organizations to the refugee crisis have led to the establishment of a complex sub-system for its utilizers. This complex system, which aims to provide financial coverage to improve access to clinical care, can be daunting for refugee women to navigate and might increase their social vulnerability during pregnancy and childbirth. Given the complexity of the Lebanese healthcare system and the multiplicity of players in it, understanding refugee women’s experiences with the use of this system within a protracted crisis situation is necessary to shed light on their perceived quality of care which may influence access and use of services. There is limited information about women’s perspectives of the dimensions of disrespect and mistreatment during childbirth in Arab countries [32, 33] and none among refugee groups who carry the burden of multiple vulnerabilities. Disrespect and mistreatment are multi-factorial and can be perceived and experienced differently depending on the context[34]. A recent review of women’s reports on obstetric violence in the Eastern Mediterranean region indicates that disrespect and abuse during childbirth is reported by women from all countries of the region and that socio-cultural norms related to patriarchy and the professional dominance of healthcare providers normalize these behaviors with the healthcare system [35]. Our previous work in Lebanon and Arab countries has shown that women are silenced and marginalized in the healthcare system during childbirth [36]. Given this background, we aim at understanding women’s interpretation of the meaning and experiences of “respect” and “disrespect”. This study aims at presenting Palestinian, Iraqi and Syrian refugee women’s experiences, understanding and interpretation of disrespect and mistreatment during childbirth in hospitals in Lebanon. A thorough understanding of these factors can guide interventions aiming at the provision of respectful maternity care to refugee women and ultimately optimize continuous access to life-saving interventions and safe essential care.

## Methods

We used a qualitative research approach which allows the researcher to obtain an insider’s view of the experiences and views of the people participating in the study [37]. Since this is an exploratory study about experiences of refugee women of their labor and birth and their interpretation of the care delivery process, qualitative research methods are appropriate to capture and explain participants’ perspectives and the meanings they attach to them [38]. We used phenomenology which allows the researcher to explore the meanings of a particular phenomenon through the personal experiences of individuals using in-depth interviews [39].

The research team consisted of academic researchers who were well versed in qualitative methodology and reproductive/maternal health, and fluent in both English and Arabic. They are not affiliated with the organizations that provide care or cover the expenses of care for the women in the study.

The recruitment of participants was carried out in two ways as a result of the national lockdown due to the COVID-19 pandemic. Face-to-face interviews were carried out before the COVID-19 outbreak between October 2019 and February 2020. Women who were 3 to 6 months postpartum were recruited through the NGOs that are actively engaged in providing welfare and healthcare services to different populations of refugee women in Lebanon. The staff of a selection of NGOs contacted eligible women (3 to 6 months after birth) in different communities where they provide social and health services, such as home visitation and humanitarian programs. They described the study using a script and a study summary which was provided by the study team. The NGO staff shared arranged appointments with women who expressed interest in participating in the study, and communicated the schedule with the research team. A trained female member of the research team met with the prospective participants according to appointments set, provided further explanation about the study and obtained their consent to participate, using an IRB approved informed consent form. The interviews were conducted either at the women’s homes or in the healthcare center run by the respective NGO. Following the national lockdown, the interviews were conducted through video and voice calls using mobile applications available to women in the period extending from May to June 2020. All interviews were conducted in Arabic, audio-recorded after the consent of the participants and transcribed verbatim by a trained transcriptionist. An interview guide of open-ended questions was developed based on the concepts used in tools suggested by Bohren et al. [1] to capture mistreatment and abuse during childbirth. The questions addressed women’s experiences and expectations with interpersonal aspects of care, their perspectives on what constitutes respectful care during childbirth, their expectations of the healthcare system, and their suggestions on how to improve the experience of care.

In total, 24 women were interviewed with variability in nationality and parity. Data collection stopped after reaching thematic saturation. Data analysis was conducted concurrently with data collection using thematic analysis [40] and following the six-stage framework suggested by Braun & Clarke [41], namely, familiarization with data, generation of initial codes, reviewing themes, defining and naming themes, and producing the final report. The researchers read the transcripts individually and then engaged in a process of open coding guided by the research aims. Matrices were constructed to explore relations between different themes and across different interviews. The research team held regular research group meetings to discuss the coding process and generation of findings through exploring recurrent and emerging themes from the data. The research team then built a consensus around the major themes and patterns emanating from the data. For their use in this paper, one of the authors translated the selected quotes into English and the others verified the translation.

## Results

Of the 24 participants in this study, 9 were Palestinian (or married to a Palestinian), 12 were Syrian and 3 were Iraqi. Their ages ranged from 19 to 38 years. Palestinian women were recruited from Tripoli and Saida (North and South of Lebanon respectively), the Iraqi women were recruited from a northern suburb of Beirut along with 3 Syrian women. The remaining Syrian women came from the Bekaa, a rural region in the east of Lebanon bordering Syria. Participants included 7 first-time mothers, 9 women who had 2 to 3 children and 8 women who had more than 4 children, including one woman with 8 children (Table 1).

**Table 1:** Characteristics of participants

Characteristics	Number of participants
**Nationality** Iraqi Palestinian Syrian	3 9 12
**Age in years** 19–24 25–29 30–34 35–39	9 3 10 2
**Number of children** 1 2–3 4–6 7–8	7 9 6 2

Despite a few positive experiences pertaining to their childbirth journey, our participants also revealed overwhelmingly adverse experiences which were obscured by their gratitude of having come out of the childbirth experience alive and safe. The adverse effects they spoke about are presented in the following themes and sub-themes: restricted choices; denial of labor companionship; life threatening and disrespectful practices with sub-standard care and mistreatment, violence and disrespect, and privacy breaches as subthemes; and money is key to good care. The way women appreciated being treated and the aspects which they considered important for being treated respectfully during childbirth in the hospital are presented in the last theme, caring and care.

### Restricted choices

Despite the positive aspects, our participants spoke about the limited options of hospitals in Lebanon for them to choose from for giving birth, as well as the limited choices of healthcare providers. The facility they ended up using was determined by where their obstetrician or midwife practices, or those contracted by UNHCR, UNRWA or NGOs which pay the bills. When they were able to choose, they relied on the reputation of the hospital from stories of other women in their communities.

“Two days before my C-section, I got a call from my doctor informing me that [hospital 1] is now approved for subsidy by the UN. She said ‘you can give birth in [hospital 1] if you want, otherwise you can in [hospital 2]’. I told her of course I want [hospital 1] because it’s better. I heard [hospital 2] doesn’t have all the medical equipment.” (Iraqi woman; 2nd child) (W11).

“I had asked for a referral to [hospital X], but they told me this referral does not work with UNRWA and they referred me to [hospital Y]. So, I gave birth in [hospital Y].” (Palestinian woman; 1st twins) (W9).

The participants’ choice of their care providers also seemed to be influenced by anecdotal evidence from other women, as well as the extent of satisfaction of the participants’ previous childbirth experiences or pre-natal care services at the primary healthcare centers.

“They [other women] advised me of him [obstetrician] as competent, comforting and with a good personality. I visited him once and then followed-up with him.” (Palestinian woman; 2nd child) (W2).

Another type of restriction is related to the decision about the type of birth, which the majority of our participants described to be in the hands of the healthcare providers, not the women themselves. While nearly all our participants reported preference for vaginal birth over cesarean section, attributing their preference to the perceived speedy recovery process, and to a lesser extent the cost, 6 of the 12 who had given birth vaginally reported that it was decided by the provider and it happened to coincide with the women’s preferences.

“The doctor examined me and told me that I had a dilated cervix and she told me I would give birth vaginally. I had initially wanted to give birth vaginally.” (Iraqi woman; 2nd child) (W14).

The small number of participants who told us they were scared of labor pain and perceived C-section as less painful were first time mothers. Some women were urged to have a C-section to avoid complications without participating in the decision-making process. In the case of one Syrian woman, the healthcare provider used fear of losing the fetus to urge the woman to have a C-section. Whether the C-section was clinically indicated or not, the use of fear restricted the choice of woman regarding the type of birth.

“I visited the obstetrician in his clinic and told him that I was in a lot of pain and that my water broke… He said that I should go to the hospital in the morning because we can’t wait longer after my water broke. He told me to choose between having a C-section or my son would die in my womb. I told him, I choose the C-section then.” (Syrian woman married to a Palestinian; 3rd child) (W7).

### Denial of labor companionship

Only 3 out of the 24 women reported they had labor companions. Many others wished for a family member to accompany them during labor and birth mentioning fear that emanates from the stories of other women or their earlier childbirth experiences. Participants who gave birth without a companion told us about their dread of childbirth pain, or of dying in the process, and of their mistrust of medical procedures in the hospital which would affect the well-being of the baby.

“Yes, I would prefer it if someone comes in with me *[pause]* because I am scared of the pain during childbirth for the first time, a lot of pain, I was in a lot of pain.” (Syrian woman; 1st child) (W19).

The majority of our participants (17 women) reported that they were denied labor companions because of hospital rules which, as three explained, also prohibited husbands from entering the delivery rooms because of their gender.

“Inside the delivery room, no, they don’t let anyone in. I go in alone.” (Syrian woman; 7th child) (W21).

“Yes, I wanted (name), my husband. They didn’t allow it. They said men are not allowed inside the operating room.” (Iraqi woman; 2nd child) (W11).

The very few participants who were allowed such companionship explained that their husbands are not prepared to witness childbirth, and therefore they were accompanied by their female relatives.

“They asked me, I said no; I mean because he gets scared.” (Iraqi woman; 2nd child) (W14).

### Life-threatening and disrespectful practices

Adverse, traumatizing and life-threatening experiences dominated women’s narratives and presented in the terms they used to describe them. A Palestinian woman for example, described the impersonal experience in the operating room “like someone who just entered a slaughterhouse”. Others spoke of wrongdoings by the healthcare team, such as inadequate medical practices, verbal and physical violence, as well as breaches of the participants’ privacy.

#### Sub-standard care and mistreatment

Among the adverse experiences reported were medical malpractice, such as errors in administering anesthesia, and in updating the patient’s medical file, unnecessary C-sections and surgery, and unnoticed internal bleeding.

“They cut me open and the anesthesia hadn’t kicked in yet. They didn’t know how to cut me; they cut me twice. Then they begged me not to file a complaint.” (Syrian woman married to a Palestinian; 3rd child) (W7).

“After I gave birth and suffered during childbirth, I got fever which happened because something wasn’t sterilized. Forty days after childbirth, I had to insert a contraceptive because I did not want to go through a similar experience. So, I used the contraceptive and on the very same day I was rushed to the hospital for a surgery to remove the contraceptive which was placed incorrectly,” (Syrian woman; 4th child) (W20).

#### Violence and disrespect

Most of the participants reported problematic verbal and non-verbal interactions with the healthcare providers. They labelled most of the verbal communication with the hospital staff as disrespectful, discriminatory, and insulting. They spoke of several instances when they felt discriminated against and subject to verbal abuse and physical violence.

“During my first birth, I witnessed nurses hitting the patients around me. They would say things like ‘oh how many children you Syrians give birth to!’” (Syrian woman; 4th child) (W20).

They described how they found themselves in conflicts with healthcare providers with disrespectful attitudes who did not empathize with women’s feelings, and prevented them from expressing their pain. Throughout these interactions, women were being blamed for expressing pain, bearing many children and for expressing their needs of holding their newborn. This process of communication nurtures feelings of guilt and being seen as an outsider.

“For example, if I ask them for a painkiller they would say, ‘we just gave you pain medication, what do you mean you’re in pain? During my first childbirth, one of them [nurse] was rude to me. She told me ‘calm down it is not very painful yet’ and examined me. I said ‘why are you shouting at me? I’m the one in pain not you!’ But when the obstetrician came, the way she [nurse] treated me changed completely.” (Palestinian woman; 2nd child) (W2).

“When I was in a lot of pain she would yell at me so that I wouldn’t scream… I mean I was screaming, so she would want me to stop to help her when I get a contraction.” (Syrian woman; 1st child) (W19).

“I felt like I was a stranger and a criminal. There I felt my soul leaving my body! Bring me my child, I want to see him! They would reply ‘in a while, later; in a while, later’.” (Syrian woman married to a Palestinian; 3rd child) (W7).

#### Privacy breaches

The interviews revealed that our participants were reluctant to talk about privacy issues, which was evident through their non-verbals during the interviews (shy, withdrawn, and speaking in a low voice). Seven participants revealed incidents of privacy violation including removing all the woman’s clothing, repeated and non-consented vaginal examination by different providers, the presence of more than one male provider in the room, and sharing delivery rooms separated by only a curtain, which they described as embarrassing and uncomfortable.

“I was shocked! There were men inside and I am veiled! As a veiled woman there should have been only one female nurse. There was one female nurse and approximately five men inside; this part I did not like. They took off my clothes suddenly and there were men and I was exposed in front of men other than my husband! So, it was a little hard for me, I mean I am conservative and religious, I don’t even shake hands with men.” (Palestinian woman; 4th child) (W5).

### Money is key to good care

Several of our participants stated that good care requires money, regardless of where it came from, and that when money is available, the care and treatment are far better. Money would provide them with access to private physicians and consequently, to perceived better hospital care. Also, if the cost of care or childbirth is covered by UN agencies and NGOs rather than the women themselves, regardless of their nationality, they are treated with disrespect, are detained, or their newborns are withheld.

“If I die at the doors [of the hospital], whether Lebanese or Syrian, I am talking about both… I mean God help the Syrian and God help the Lebanese, we are both in the same situation: a person who does not have money does not enter (the hospital).” (Syrian woman; 2nd child) (W15).

“A nurse told me ‘if you want, we can relieve all this pain you’re feeling… with an injection which costs $100’.” (Palestinian woman; 3rd child) (W6).

Two participants pointed to disrespectful treatment in the discharge process: being kicked out or withholding the baby because of unpaid hospital bills or until they paid them.

“My son! They took my son! You know? They kept him in the room until I paid the bill, then they gave him to me. They thought we would run away!” (Palestinian woman; 4th child) (W5).

“You feel as if they are kicking you out. I told them remove my I.V., I can sit outside in front of you if you would like to let another patient take my room. I felt like I was bothering them by being in the room (while waiting for my bill to be paid).” (Palestinian woman; 3rd child) (W6).

### Caring and care

The aspects of childbirth experiences that our participants described as positive include the cleanliness of the facility, attention to the mother’s and her newborn’s personal hygiene, the availability of food in the hospital, television and music.

“When it’s time for the birth, she [nurse] stays with you until you’re done; when the baby comes, they clean him and change his clothes; the hospital it is very, very clean.” (Iraqi woman; 2nd child) (W12).

The participants articulated what makes them feel valued. They appreciated responsiveness with support, and caring attitudes of healthcare providers; and to a lesser extent being physically comfortable and having access to pain relief. They gave examples, such as frequent check-ups by healthcare providers, caring for the baby, reassurance, respectful communication including refraining from using foul language, holding a woman’s hand and reading the Koran or the Bible to her, saying a prayer, paying attention to their personal hygiene, and cleaning the hospital room.

“For instance, when you are in pain after childbirth and are having cramps, you tell them you’re in pain, they bring medicine and put it in the I.V. You feel like someone is helping you, and is responding to you. Also, you feel appreciated once they clean your bed and put new sheets.” (Syrian woman; 7th child) (W 21).

The participants spoke about what they perceived as admirable attributes of the obstetricians, midwives and nurses they interacted with. The majority described midwives as supportive, comforting and soothing. These attributes were not assigned to obstetricians who were predominantly male. They pointed out certain characteristics of midwives, such as being supportive, not complaining, having a comforting touch, safeguarding their privacy, and being empathetic and reassuring. They described these qualities as helpful in relieving their physical pain.

“With the obstetrician, there was a midwife who was also Syrian, she helped me a lot. Maybe she had a lot of experience since she is older. She helped me a lot, ‘do this, do that; don’t go up, don’t go down, don’t move’… She would hold my hand, things that you might not imagine would happen. If you have someone by your side it would help. The midwife being there was comforting.” (Syrian woman; 2nd child) (W13).

Only 3 participants reported being fully supported by midwives while giving birth. The midwives took over providing care when the woman’s obstetrician was not on call at the time. All 3 reported positive experiences with midwives, stating their preference for a female provider, which was not always the case.

“There would be a midwife and someone helping her; they are very, very nice… they comfort you and give you confidence, they are patient and they read a lot of Koran.” (Palestinian woman; 3rd child) (W6).

## Discussion

This study is the first to explore refugee women’s understanding of respectful maternity care and their interpretation of disrespect and abuse in Lebanese hospitals. Whereas research efforts measuring disrespect, mistreatment and abuse during childbirth have focused on quantifying the burden of mistreatment and abuse for women in different contexts [1, 42], this study has captured the multiple meanings of respectful care for refugee women and provided an in-depth understanding of it. Our findings contribute to the scarce research which documents experiences with childbirth among conflict affected populations [43], and sheds light on the experiences of a specific group labeled as refugees despite their different experiences with displacement. Their experiences with mistreatment and abuse however, are very similar and are related to their refugee status rather than to their years of residence in Lebanon (Palestinian women born in Lebanon to refugee families vs. Iraqi and Syrian women arriving to Lebanon as refugees), and are indicative of intersecting marginalization with their economic status.

The types of mistreatment and abuse described by the women in this study align with the domains recognized in the literature [1, 42]. These refugee women who are from different nationalities giving birth in hospitals in various regions in Lebanon are subject to verbal abuse through judgmental comments and the use of harsh language by healthcare providers as they endure poor staff attitudes shaped by the discriminatory and stigmatizing perception about refugees [26]. The care they received fails to meet professional standards: denial of pain relief options or performance of painful surgical procedures, being subject to objectification through lack of respect for cultural preferences and lack of privacy. Our findings also resonate with global evidence on women’s preference for labor companions [44] and previous research from Lebanon where women, including refugees, expressed their fear from laboring alone in hospitals, and their need to have a female family member accompanying them during childbirth [23]. These findings agree with the WHO framework where experience of care represented by the domains of effective communication, respect and dignity, and emotional support, is considered as an integral part of quality of care [3]. Our findings indicate that women present their experiences with disrespectful care on the interpersonal level and with the sub-standard clinical care they received, as inter-related aspects of mistreatment and abuse.

Our study found that refugee women are unable to make choices about the birthing facilities, providers, or type of birth without restrictions because the physicians and the payer parties are the ones who make those decisions. Our findings show that navigating this and other formal societal institutions is facilitated by the social networks which connect the women and other community members in collectivist Arab societies. Although the WHO framework focuses on facility-based care, it acknowledges the importance of community engagement and the role of families and the social network of women in accessing quality childbirth care [3]. In collectivist cultures, social relationships are dense and manifest through tribal, immediate and extended family networks and beyond [45]. This connectivity ensures access to information, as well as cohesion and survival [46]. It extends to serve refugee populations in diaspora and in their host countries [47] and provides refugee women with various forms of social support.

The value of the support imparted by this collectivist society is not sufficiently embedded in the health system used by refugee women. The very structures that are meant to support women in childbirth are making their experiences more difficult, be it from the shortfalls in care at the health facility or through the humanitarian aid system. The fear from medical procedures and from being subject to sub-standard care expressed in women’s narratives reflects the lack of accountability in the Lebanese healthcare system [48] especially when they consider they are reliant on humanitarian aid to receive healthcare because they are refugees. These systems continue to alienate and silence women, deprive them of their right to information, segregate them as refugees and isolate them from their support systems [49]. Similarly, women’s choices of their healthcare providers, type of birth and anesthetic are limited. This finding stretches the domains of availability of competent human resources and essential physical resources in the WHO framework of quality of care [3] to incorporate restricted choices in access to quality of care for refugee communities. Women emphasized the importance of out-of-pocket payments in the predominantly privatized Lebanese healthcare system to overcome these restrictions which exemplify the social exclusionary policies and practices that women experience at multiple levels therein [50]. Women are forced into a reality unarmed with information and dominated by fear, a situation that can adversely impact their childbirth experiences and the well-being of the women and their newborns.

The women in this study have low expectations of the healthcare system and they report feeling pampered for having what should be their basic rights during facility-based births, such as privacy, respect, pain relief, clean sheets and food. These findings concur with the literature on women’s childbirth expectations which is pre-conceived through their previous experiences and through what is offered by the healthcare services for their communities [51]. This study shows that for these refugee women, their “silent endurance” of mistreatment and abuse in this system is not only a reflection of similar lived experiences in a society where they are marginalized but also an expectation from being the recipient of humanitarian aid. This in turn is normalized in the organization of the healthcare system, which is not engaging women but rather reproducing their marginalization. All of this leads to disempowerment of women and the consequent low expectations they have of care provision which leads to normalization of disrespectful care [13].

This study provides a number of implications for further research, for policy and practice that can promote positive experiences for women experiences with maternal healthcare in humanitarian contexts. Investing more in research that captures the multiple dimensions of the meaning of disrespect and abuse will provide the necessary in-depth understandings that inform implementation research and interventions at the health system level in contexts affected by conflict. Considering refugee women’s perspectives, values and cultural preferences in the organization and delivery of healthcare in conflict affected populations is key. Mechanisms for the inclusion of women in the organization and delivery of maternal healthcare need to be developed to enhance accountability within the system. Humanitarian responses need to capitalize on collectivist societal values and use community assets to mobilize female healthcare practitioners especially those from within the refugee populations and to preserve women’s rights to benefit from labor and birth companionship. Integration of subjects that emphasize cultural competence and empathy is a recommended change in medical education and training in addition to the need to have more midwives practicing independently in healthcare facilities in Lebanon. Considering that humanitarian sub-systems restrict women’s choices and have failed to provide the appropriate and respectful care that they are intended for during crises, a different approach to healthcare cost coverage is thus necessary to meet the needs of refugee women living in protracted crises.

The limitations in this study are as follows. There was no information on the registration status of the women as refugees. Although there are no large variations in the financial coverage of birthing care between registered and unregistered refugees, however there might have been slight variations in the experiences of navigating the system which was not captured in this study. The study also did not include the experiences of impoverished Lebanese women who are also enduring multiple vulnerabilities for comparison. Women’s subjective accounts of their childbirth experiences may be influenced by normalization of disrespect and abuse especially among those who had healthy newborns and did not suffer from major complications.

## Conclusion

Through the use of qualitative methodology, our study contributes with new knowledge by shedding light on the meaning of respectful care in the context of a system that was created specifically to serve the needs of refugee populations, a system that has proven through the narratives of refugee women to be equally harmful.

Considering that constraints in resources are identified as contributing factors to disrespect and abuse during childbirth in different contexts [1, 50], we anticipate the aggravation of this problem both for refugee and host populations in contexts of protracted conflict and multiple crises. Innovative and multidimensional interventions are thus needed at the level of the health system to protect women’s rights to respectful maternity care. Programs and policies should address strengthening capacity and providing resources for the adaptation of global guidelines into context-specific strategies to provide quality maternity care during humanitarian crises and beyond.

## Electronic supplementary material

Below is the link to the electronic supplementary material.

Supplementary Material 1

Supplementary Material 2

## Data Availability

The datasets generated and/or analyzed during the current study are not publicly available to protect participants’ privacy but are available from the corresponding author on reasonable request.
