# Supplementary material for: “A person who does not have money does not enter”: a qualitative study on refugee women’s experiences of respectful maternity care
Source: BMC Pregnancy Childbirth. 2022 Oct 5;22:748. doi: 10.1186/s12884-022-05083-2 (PMC9533279; doi:10.1186/s12884-022-05083-2)
Supplement: Supplementary file 1 — Supplementary Material 1 [file 12884_2022_5083_MOESM1_ESM.docx]

**“A person who does not have money does not enter": a qualitative study on refugee women’s experiences of respectful maternity care**

**Interview guide: Women**

**Introduction:** Thank you for agreeing to participate in the study. As mentioned in the consent form, all information discussed is confidential and will not be disclosed to anyone. Also, please do not state any sensitive information or any information that may identify specific incidents/individuals. You may stop at any point if you feel uncomfortable or if you change your mind.

1. Congratulations on the birth of your baby. How long has it been since you gave birth? Tell me a little bit about your family. How many children do you have now? Who is living with you in this house? How long have you been in Lebanon?
2. Where did you give birth? How did you choose that hospital/clinic in particular?
3. Tell me about your experience of giving birth at that hospital/clinic.

a) What did you like most about that experience?

b) What bothered you most about that experience?

Probes: relationship with staff (obstetrician, nurses, midwives, others)? How was the communication between you and the team taking care of you?

1. What expectations did you have during labor and birth? Were you given these options? Tell me how? Can you tell me what happened/give me an example? Tell me how did you feel about that?
2. What can you say about the treatment given to you as a non-Lebanese? How did you feel about that? Can you give me an example?
3. If you were to give birth again in that hospital/clinic, what would you want to be different? What are specific things that would make you feel respected/valued? What would you look for in the care provided during labor, birth and throughout your stay at the hospital?
