# Supplementary material for: “A person who does not have money does not enter”: a qualitative study on refugee women’s experiences of respectful maternity care
Source: BMC Pregnancy Childbirth. 2022 Oct 5;22:748. doi: 10.1186/s12884-022-05083-2 (PMC9533279; doi:10.1186/s12884-022-05083-2)
Supplement: Supplementary file 2 — Supplementary Material 2 [file 12884_2022_5083_MOESM2_ESM.docx]

**“A person who does not have money does not enter": a qualitative study on refugee women’s experiences of respectful maternity care**

**Major Categories and codes**

| **Categories** | **Codes** |
| --- | --- |
| Financial Considerations | For access to care  For Quality of care  For Detention in facility |
| Choice of hospital |  |
| Choice of health care provider | Obstetrician  Midwife |
| Choice in pain relief |  |
| Experience with pain relief |  |
| Experiences with health care providers | With obstetricians  With Midwives  With Nurse  With Anesthesiologist |
| Other women’s childbirth experiences |  |
| Availability of health care providers |  |
| Privacy concerns |  |
| Expectations about childbirth experience |  |
| Comparing services | By nationality  By refugee status |
| Women’s sense of appreciation (being valued) |  |
| Evaluation of hospital setting | Public hospitals  Private hospitals |
| Perceived clinical errors |  |
| Evaluation of the overall experience | Best aspects of care  Worst aspects of care |
| Labor companionship | Role of husband  Role of companion  Need and expectations  Experiences |
